# Supplementary material for: A strain of Streptococcus mitis inhibits biofilm formation of caries pathogens via abundant hydrogen peroxide production
Source: Appl Environ Microbiol. 2025 Feb 25;91(3):e02192-24. doi: 10.1128/aem.02192-24 (PMC11921374; doi:10.1128/aem.02192-24)
Supplement: Supplemental material — Figures S1 to S3; Tables S1 to S9. [file aem.02192-24-s0002.pdf]

Supplemental Material

A Strain of *Streptococcus mitis* Inhibits Biofilm Formation of Caries Pathogens via Abundant Hydrogen Peroxide Production

Isabella Williams*, Jacob S. Tuckerman*, Daniel I. Peters, Madisen Bangs, Emily Williams, Iris J. Shin, and Justin R. Kaspar [#]

Division of Biosciences, The Ohio State University College of Dentistry, Columbus, Ohio

Table of Contents:

Supplemental Figures (S1-S3): Pages 2 - 4

Supplemental Tables (Table S1-S9): Pages 5 – 27

Supplemental Excel File: Contains analyzed RNA-Seq data (log2 fold change and –log10 P values) for each gene within either indicated species, used to construct Supplemental Tables 1-4.

* Co-first authors
[#] Corresponding author

Mailing address:
Division of Biosciences, The Ohio State University, College of Dentistry,
305 W. 12th Avenue, Postle Hall Rm 4185, Columbus, OH 43210.
Phone: (614) 292-3373
E-mail: kaspar.17@osu.edu

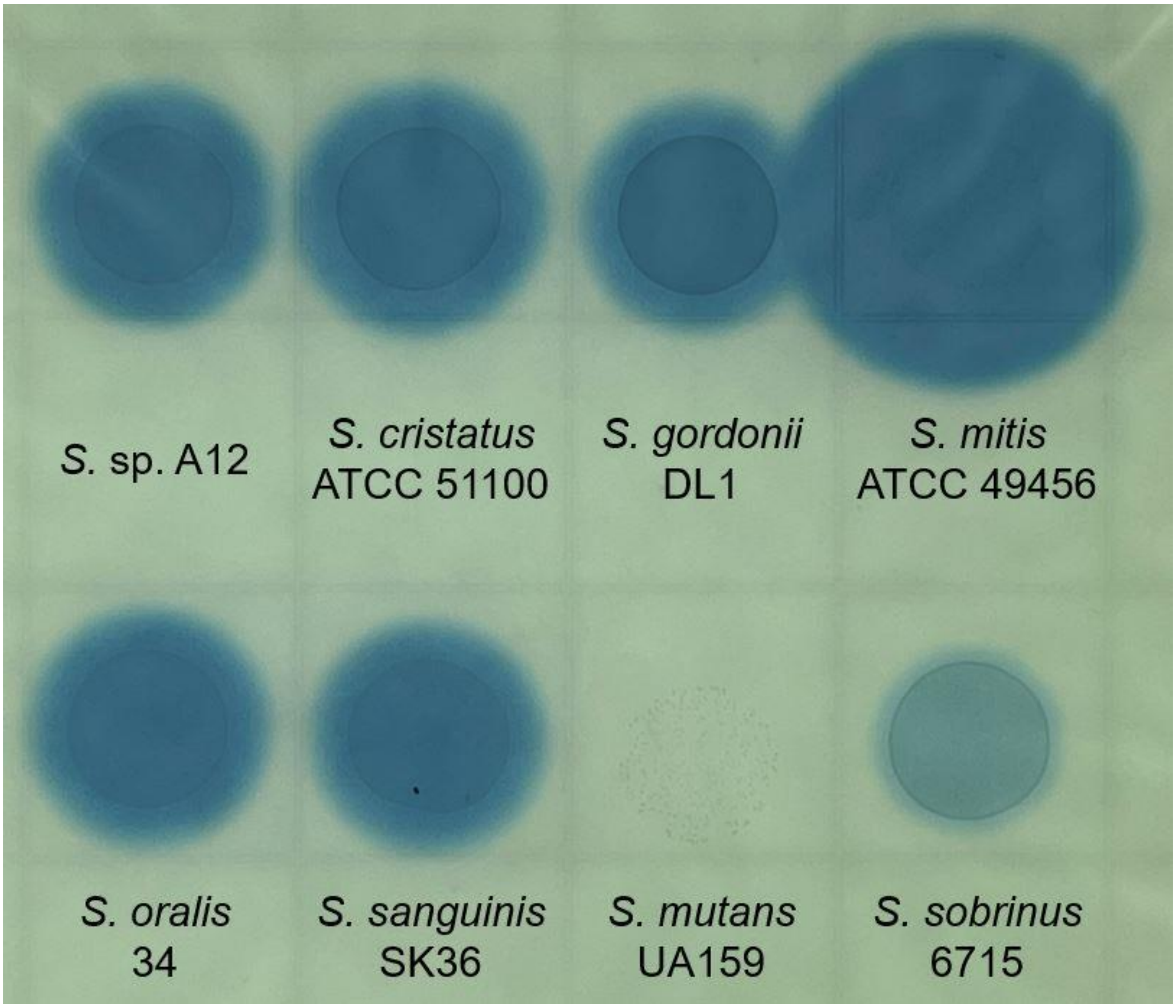

Supplemental Figure 1. Prussian blue agar assay for hydrogen peroxide detection in different *Streptococcus* species. Eight different species of oral streptococci were spotted (10 µL of OD600 nm = 0.1 cell suspension) on Prussian blue agar plates and incubated for 48 h at 37°C and 5% CO_2. The blue around each individual spotted colony represents hexacyanoferrate (III) and iron (III) present within the agar plates that precipiates in the presence of hydrogen peroxide. This experiment shows increased hydrogen peroxide production by *S. mitis* ATCC 49456 in comparison to other oral streptococci tested.

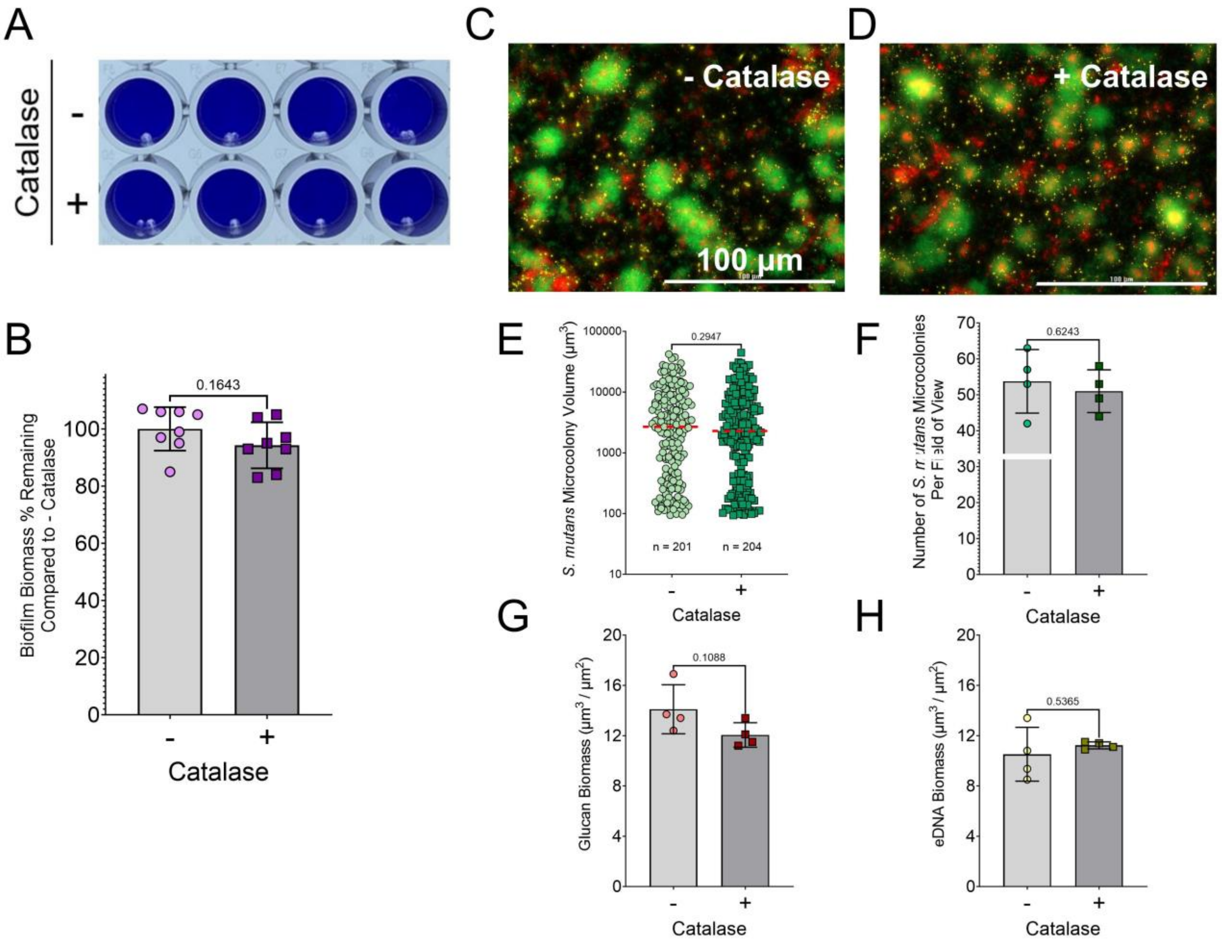

Supplemental Figure 2. *Streptococcus mutans* monoculture biofilms in the presence and absence of 100 U mL^{-1} catalase. **(A)** Representative image of a CV biofilm biomass assay where *S. mutans* is cultured with (+) or without (-) 100 U mL^{-1} catalase. **(B)** CV quantification (Abs 575 nm) of the experiment shown in A. Data is expressed as the percentage of biomass formed in comparison to *S. mutans* monoculture without catalase addition (i.e., - catalase values set to 100%). n = 8. **(C)** Merged representative maximum intensity 40x Z-projection of 24 h *S. mutans* monoculture biofilm grown in TYGS medium without catalase. *S. mutans* constitutively expresses GFP (green), eDNA was probed with labeled antibodies (yellow), and glucans visualized with labeled dextran (red). Scale bar (100 µm) is shown in the bottom right corner. **(D)** Merged representative maximum intensity 40X Z-projection of 24 h *S. mutans* monoculture biofilm grown in TYGS medium with addition of 100 U mL^{-1} catalase. **(E)** Quantification of individual *S. mutans* microcolony volumes, **(F)** number of *S. mutans* microcolonies per field of view, **(G)** glucan biomass, and **(H)** eDNA biomass from the microscopy data shown between the two conditions. n = 4. Quantification was completed using Gen5 Image+ software. Data graphing and one-way analysis of variance (ANOVA) with multiple comparisons was completed in GraphPad Prism software. Data shows no significant differences in *S. mutans* biofilm biomass composition in the presence of catalase.

```
promoter_spxB_mitis_b6        -------tgactagttcactcttactaaactttagtaaaata---gactaaaagcgggct   50
promoter_spxB_mitis_49456     tgcaacctcaaagctgtgctttgagcaacctgtggcgagcttcctagtttgctctgtgat   60
                                     * *    *   ** * *  ** ** * *  *  *       *      * * *

promoter_spxB_mitis_b6        tgctcgcttttattataaaacagttctgtaaaacgctttcggactgtactgtaatagaga   110
promoter_spxB_mitis_49456     tttcattgagtattataaaacagaagctaaaaacgttttcaaactgtactgtaatagaga   120
                              *         *************      ****** ****  ******************

promoter_spxB_mitis_b6        atagaaacttctgagcagattcttagaaaagtcgaacgaaatgtgataaattagtagagt   170
promoter_spxB_mitis_49456     ataaaaacttctgagcagattcttagtaaagtcaaaataaatgtgataaattagtattgt   180
                              *** ********************** ****** **  ******************  **

promoter_spxB_mitis_b6        aaaaatttgctgaaacgttttcaaaaactatatgaaacttt-tttagtagatttaaaaat   229
promoter_spxB_mitis_49456     aaaaatttactgaaacgttttcaaaaactatatgaaacctttttagtagatttaaaaat   240
                              ******** ***************************** ** ******************
                                   RBS              Start of ORF
promoter_spxB_mitis_b6        atttgaaggagagttatcattATG 253
promoter_spxB_mitis_49456     atttgaaggagagttatcattATG 264
                              ************************
```

Supplemental Figure 3. ***spxB*** **promoter alignment between** ***Streptococcus mitis*** **B6 and** ***Streptococcus mitis*** **ATCC 49456.** Alignment of 250 bp upstream of the *spxB* start site (ATG) in *S. mitis* B6 and 261 bp upstream in *S. mitis* ATCC 49456. The 3' end of the sequence represents the site closest to the start codon for *spxB*. The ribosome binding site (RBS) has been underlined. Alignment was completed with the Clustal Omega multiple sequence alignment (MSA) tool with default settings.

SUPPLEMENTAL TABLES

Supplemental Table 1. Differentially expressed genes* (DEGs) between *Streptococcus mitis* ATCC 49456 grown in monoculture vs coculture with *Streptococcus mutans* UA159**.** Red, upregulated genes. Blue, downregulated genes.

*Differentially expressed genes = genes with $\geq$4 Log10 *P* value and Log2 fold change (FC) $\geq$ (-)1.

Gene ID	Gene Name	Gene Description	Log2 Fold Change	Fold Change	P value	Log10 P value
SM12261_RS02695		sugar ABC transporter permease	1.98	3.94	1.47E-15	14.8
SM12261_RS02690		ABC transporter substrate-binding protein	1.89	3.69	1.16E-20	19.9
SM12261_RS09385			1.80	3.48	9.09E-09	8.0
SM12261_RS02700		carbohydrate ABC transporter permease	1.73	3.33	7.93E-11	10.1
SM12261_RS01035	adhE	bifunctional acetaldehyde-CoA/alcohol dehydrogenase	1.62	3.08	4.26E-16	15.4
SM12261_RS01275	adhP	alcohol dehydrogenase AdhP	1.55	2.94	1.52E-16	15.8
SM12261_RS08975		ABC transporter substrate-binding protein	1.45	2.74	2.45E-10	9.6
SM12261_RS01820		CPBP family intramembrane metalloprotease	1.40	2.65	7.63E-11	10.1
SM12261_RS06095	pdxT	pyridoxal 5'-phosphate synthase glutaminase subunit PdxT	1.39	2.62	5.67E-18	17.2
SM12261_RS02705		YhcH/YjgK/YiaL family protein	1.36	2.57	7.61E-08	7.1
SM12261_RS05425		sugar ABC transporter ATP-binding protein	1.34	2.53	2.51E-10	9.6
SM12261_RS01815		hypothetical protein	1.26	2.39	1.81E-08	7.7
SM12261_RS06100	pdxS	pyridoxal 5'-phosphate synthase lyase subunit PdxS	1.25	2.38	3.11E-15	14.5
SM12261_RS07895		helix-turn-helix transcriptional regulator	1.19	2.29	1.74E-07	6.8
SM12261_RS08950		hypothetical protein	1.18	2.27	6.43E-11	10.2
SM12261_RS04745	dhaL	dihydroxyacetone kinase subunit L	1.18	2.26	8.49E-12	11.1
SM12261_RS08965		bacteriocin immunity protein	1.17	2.25	1.49503E-05	4.8
SM12261_RS02685		PTS transporter subunit EIIC	1.17	2.25	3.29E-10	9.5
SM12261_RS09150		hypothetical protein	1.17	2.24	2.64E-17	16.6
SM12261_RS05415		substrate-binding domain-containing protein	1.17	2.24	2.87E-10	9.5
SM12261_RS00935		zinc-dependent alcohol dehydrogenase family protein	1.16	2.24	3.09E-13	12.5
SM12261_RS05430	rbsD	D-ribose pyranase	1.15	2.21	1.25752E-05	4.9
SM12261_RS05420	rbsC	ribose ABC transporter permease	1.14	2.21	6.07258E-06	5.2
SM12261_RS05225		glucose-1-phosphate adenylyltransferase	1.12	2.18	2.40E-11	10.6
SM12261_RS04750		PTS-dependent dihydroxyacetone kinase phosphotransferase subunit DhaM	1.10	2.15	1.0872E-06	6.0
SM12261_RS00630		sugar ABC transporter permease	1.10	2.15	7.75E-07	6.1

SM12261_RS07200	ugpC	sn-glycerol-3-phosphate ABC transporter ATP-binding protein UgpC	1.10	2.14	2.2392E-05	4.6
SM12261_RS08945		DUF4097 family beta strand repeat-containing protein	1.03	2.05	1.95573E-06	5.7
SM12261_RS06215		endonuclease/exonuclease/phosphatase family protein	1.03	2.04	1.75E-07	6.8
SM12261_RS09375		competence system sensor histidine kinase ComD	-1.09	-2.12	1.97E-11	10.7
SM12261_RS06165		bacteriocin immunity protein	-1.10	-2.15	1.15375E-05	4.9

Supplemental Table 2. DEGs between *Streptococcus mutans* UA159 grown in monoculture vs coculture with *Streptococcus mitis* ATCC 49456**.** Red, upregulated genes. Blue, downregulated genes.

Gene ID	Gene Name	Gene Description	Log2 Fold Change	Fold Change	P value	Log10 P value
**SMU_2027**		transcriptional regulator/repressor	3.70	13.04	8.73E-07	6.1
**SMU_1470c**		conserved hypothetical protein	3.61	12.22	1.61E-09	8.8
**SMU_202c**		conserved hypothetical protein/Streptococcus-specific protein	3.40	10.56	2.24E-06	5.7
**SMU_206c**		hypothetical protein	3.25	9.54	1.31E-19	18.9
**SMU_1888**		transposase fragment	3.25	9.50	6.37E-18	17.2
**SMU_210c**		hypothetical protein	3.24	9.43	1.30E-14	13.9
**SMU_1752c**		hypothetical protein	3.21	9.24	1.60E-14	13.8
**SMU_207c**		transcriptional regulator	3.15	8.88	1.59E-16	15.8
**SMU_1764c**		conserved hypothetical protein	3.14	8.82	6.55E-08	7.2
**SMU_1762c**		conserved hypothetical protein	3.14	8.79	3.82E-14	13.4
**SMU_200c**		hypothetical protein	3.11	8.60	6.72E-12	11.2
**SMU_2104a**		50S ribosomal protein L32	3.08	8.44	4.04E-17	16.4
**SMU_211c**		hypothetical protein	3.07	8.43	1.55E-17	16.8
**SMU_1753c**		conserved hypothetical protein	3.05	8.26	2.83E-11	10.5
**SMU_1763c**		conserved hypothetical protein	3.03	8.16	7.08E-15	14.2
**SMU_714**		translation elongation factor Tu	3.03	8.15	8.49E-09	8.1
**SMU_201c**		conserved hypothetical protein	3.01	8.08	1.77E-12	11.8
**SMU_1754c**		conserved hypothetical protein	2.99	7.93	5.82E-08	7.2
**SMU_208c**		conserved hypothetical protein, FtsK/SpoIIIE family	2.98	7.90	3.46E-09	8.5
**SMU_1760c**		conserved hypothetical protein	2.98	7.87	7.00E-11	10.2
**SMU_1247**	*eno*	enolase	2.93	7.62	3.82E-06	5.4
**SMU_1758c**		conserved hypothetical protein	2.87	7.34	2.54E-09	8.6
**SMU_1755c**		conserved hypothetical protein	2.86	7.26	1.56E-13	12.8
**SMU_1750c**		hypothetical protein	2.84	7.14	1.40E-07	6.9
**SMU_360**	*gapC*	glyceraldehyde-3-phosphate dehydrogenase; plasmin receptor	2.82	7.05	1.80E-08	7.7
**SMU_2026c**		30S ribosomal protein S10 fragment	2.81	7.01	2.70E-16	15.6
**SMU_209c**		hypothetical protein	2.80	6.97	2.09E-11	10.7
**SMU_1757c**		conserved hypothetical protein	2.76	6.76	1.74E-11	10.8
**SMU_1761c**		conserved hypothetical protein	2.75	6.72	1.01E-13	13.0
**SMU_213c**		hypothetical protein	2.66	6.33	1.17E-14	13.9
**SMU_99**	*fbaA*	fructose-bisphosphate aldolase	2.64	6.25	6.92E-15	14.2
**SMU_205c**		conserved hypothetical protein	2.64	6.25	5.38E-15	14.3
**SMU_1682c**		conserved hypothetical protein (possible intracellular protease)	2.61	6.09	5.82E-14	13.2
**SMU_198c**		conjugative transposon protein	2.60	6.05	1.01E-10	10.0
**SMU_215c**		hypothetical protein	2.50	5.65	1.56E-12	11.8
**SMU_212c**		hypothetical protein	2.48	5.56	4.57E-10	9.3

**SMU_204c**		hypothetical protein	2.44	5.43	6.12E-14	13.2
**SMU_199c**		hypothetical protein	2.44	5.41	1.02E-14	14.0
**SMU_849**		50S ribosomal protein L27	2.41	5.33	1.19E-10	9.9
**SMU_698**		50S ribosomal protein L35	2.33	5.01	6.58E-11	10.2
**SMU_1858**	*rs18*	30S ribosomal protein S18	2.27	4.81	1.60E-08	7.8
**SMU_402**	*pfl*	pyruvate formate-lyase	2.26	4.78	5.06E-11	10.3
**SMU_197c**		hypothetical protein	2.22	4.66	2.93E-11	10.5
**SMU_2020**	*rl16*	50S ribosomal protein L16	2.21	4.62	1.47E-13	12.8
**SMU_120**		50S ribosomal protein L28	2.20	4.58	4.55E-15	14.3
**SMU_216c**		hypothetical protein	2.18	4.53	2.68E-13	12.6
**SMU_1379**			2.17	4.51	2.96E-09	8.5
**SMU_2010**	*rl18*	50S ribosomal protein L18	2.16	4.46	1.09E-13	13.0
**SMU_961**		macrophage infectivity potentiator-related protein	2.13	4.39	4.14E-16	15.4
**SMU_92c**			2.11	4.30	1.77E-14	13.8
**SMU_766**		transposase	2.06	4.17	4.51E-10	9.3
**SMU_93c**			2.04	4.12	1.41E-09	8.9
**SMU_1627**	*rl11*	50S ribosomal protein L11	1.98	3.94	4.90E-12	11.3
**SMU_1781**		conserved hypothetical protein	1.91	3.76	6.62E-14	13.2
**SMU_636**		glucosamine-6-phosphate isomerase	1.91	3.76	1.04E-06	6.0
**SMU_962**		acyl-CoA dehydrogenase	1.91	3.75	5.19E-19	18.3
**SMU_2002**	*rs11*	30S ribosomal protein S11	1.88	3.68	7.09E-13	12.1
**SMU_1782**		conserved hypothetical protein	1.81	3.50	1.50E-11	10.8
**SMU_196c**		immunogenic secreted protein (transfer protein)	1.80	3.49	4.38E-11	10.4
**SMU_936**		amino acid ABC transporter, ATP-binding protein	1.76	3.39	1.62E-05	4.8
**SMU_94c**		transposase fragment	1.65	3.13	1.22E-09	8.9
**SMU_934**		amino acid ABC transporter, permease protein	1.64	3.12	1.06E-13	13.0
**SMU_865**		30S ribosomal protein S16	1.62	3.08	1.35E-19	18.9
**SMU_1860**	*rs6*	30S ribosomal protein S6	1.61	3.04	2.43E-10	9.6
**SMU_2017**	*rl14*	50S ribosomal protein L14	1.60	3.03	2.80E-15	14.6
**SMU_1183**	*mtlA2*	phosphotransferase system enzyme II	1.60	3.02	1.78E-16	15.7
**SMU_753**		conserved hypothetical protein	1.56	2.95	1.42E-09	8.8
**SMU_997**		inorganic ion ABC transporter,ATP-binding protein; possible ferrichrome transport system	1.55	2.93	1.39E-13	12.9
**SMU_699**		50S ribosomal protein L20	1.54	2.92	6.69E-13	12.2
**SMU_1072c**		acyltransferase	1.53	2.88	6.86E-11	10.2
**SMU_589**		histone-like DNA-binding protein	1.53	2.88	8.79E-15	14.1
**SMU_2021**	*rs3*	30S ribosomal protein S3	1.52	2.88	1.17E-15	14.9
**SMU_935**		amino acid ABC transporter, permease protein	1.51	2.85	1.33E-16	15.9
**SMU_528c**		conserved hypothetical protein	1.49	2.82	9.34E-17	16.0
**SMU_940c**		hemolysin III-related protein	1.49	2.81	3.88E-14	13.4
**SMU_995**		ferrichrome ABC transporter (permease)	1.49	2.81	7.93E-11	10.1

**SMU_981**	*bglB1*	beta-glucosidase, BglB protein	1.48	2.80	8.67E-18	17.1
**SMU_217c**		conserved hypothetical protein; Streptococcus-specific protein	1.48	2.78	6.54E-12	11.2
**SMU_933**		amino acid ABC transporter, amino acid substrate-binding protein	1.47	2.76	5.30E-17	16.3
**SMU_1343c**		polyketide synthase	1.46	2.75	2.08E-16	15.7
**SMU_2016**	*rl24*	50S ribosomal protein L24	1.45	2.73	1.45E-17	16.8
**SMU_1184c**		transcriptional regulator	1.45	2.72	1.01E-09	9.0
**SMU_1182**	*mtlD*	mannitol 1-phosphate 5-dehydrogenase	1.42	2.68	7.07E-17	16.2
**SMU_1988c**		probable DNA binding protein	1.42	2.67	2.92E-12	11.5
**SMU_1341c**		gramicidin S synthase/mycosubtilin synthetase chain mycB	1.40	2.64	7.07E-18	17.2
**SMU_527**		conserved hypothetical protein	1.40	2.63	2.41E-11	10.6
**SMU_1346**	*bacT*	thioesterase II-like protein	1.40	2.63	4.37E-11	10.4
**SMU_1340**	*bacA2*	bacitracin synthetase 1/ tyrocidin synthetase III	1.39	2.62	2.11E-17	16.7
**SMU_2025**	*rl3*	50S ribosomal protein L3	1.38	2.60	7.51E-15	14.1
**SMU_998**		ABC transporter, ferrichrome-binding protein	1.38	2.60	1.01E-16	16.0
**SMU_1068c**		ABC transporter, ATP-binding protein	1.36	2.57	2.31E-08	7.6
**SMU_1345c**		peptide synthetase similar to mycA	1.33	2.51	3.75E-10	9.4
**SMU_2057c**		cadmium-efflux ATPase, E1-E2 (heavy metal-transporting ATPase)	1.32	2.50	5.03E-17	16.3
**SMU_1335c**		enoyl-acyl carrier protein(ACP) reductase; dioxygenase related to 2-nitropropane dioxygenase	1.30	2.46	1.36E-15	14.9
**SMU_1743**	*acp*	acyl carrier protein	1.25	2.38	5.53E-13	12.3
**SMU_1347c**			1.25	2.38	6.12E-11	10.2
**SMU_2019**	*rl29*	50s ribosomal protein L29	1.24	2.37	3.51E-15	14.5
**SMU_195c**		hypothetical protein	1.24	2.36	1.91E-11	10.7
**SMU_1365c**			1.22	2.33	7.84E-10	9.1
**SMU_1342**	*bacA1*	bacitracin synthetase	1.21	2.31	6.93E-10	9.2
**SMU_184**	*sloC*	ABC transporter element, iron predicted binding protein	1.20	2.30	1.07E-08	8.0
**SMU_1339**	*bacD*	bacitracin synthetase; surfactin synthetase	1.18	2.27	7.43E-17	16.1
**SMU_1344c**		malonyl CoA-acyl carrier protein transacylase	1.18	2.26	1.31E-14	13.9
**SMU_924**	*tpx*	thiol peroxidase	1.17	2.26	1.66E-10	9.8
**SMU_194c**		conserved hypothetical protein, phage-related	1.17	2.25	3.06E-08	7.5
**SMU_2022**	*rl22*	50S ribosomal protein L22	1.17	2.25	1.09E-18	18.0
**SMU_193c**		conserved hypothetical protein	1.15	2.23	3.88E-09	8.4
**SMU_1336**	*pksD*	conserved hypothetical protein	1.15	2.23	4.15E-11	10.4
**SMU_1626**	*rl1*	50S ribosomal protein L1	1.15	2.22	3.50E-10	9.5
**SMU_186**	*sloR*	metal-dependent transcriptional regulator (probable DtxR homolog)	1.15	2.22	1.15E-15	14.9
**SMU_925**		bacteriocin immunity protein	1.14	2.21	9.77E-13	12.0
**SMU_941c**		conserved hypothetical protein	1.14	2.20	6.71E-13	12.2
**SMU_185**		hypothetical protein	1.14	2.20	6.22E-07	6.2

**SMU_637c**		hypothetical protein	1.13	2.19	1.3704E-06	5.9
**SMU_769**		conserved hypothetical protein	1.12	2.18	2.14E-11	10.7
**SMU_996**		ABC transporter, permease protein;possible ferrichrome transport system	1.12	2.17	4.79E-13	12.3
**SMU_2009**	*rs5*	30S ribosomal protein S5	1.11	2.16	1.33E-16	15.9
**SMU_540**	*dpr*	peroxide resistance protein / iron binding protein	1.10	2.14	1.62E-14	13.8
**SMU_2018**	*rs17*	30S ribosomal protein S17	1.10	2.14	1.48E-17	16.8
**SMU_1337c**		alpha/beta superfamily hydrolases	1.09	2.13	2.02E-09	8.7
**SMU_154**		30S ribosomal protein S15	1.09	2.13	7.39E-12	11.1
**SMU_361**	*pgk*	phosphoglycerate kinase	1.09	2.13	1.93E-07	6.7
**SMU_358**		30S ribosomal protein S7	1.09	2.13	7.68E-18	17.1
**SMU_1366c**			1.08	2.12	7.70E-13	12.1
**SMU_1680c**		hypothetical protein	1.08	2.11	9.47E-10	9.0
**SMU_05**		conserved hypothetical protein	1.04	2.06	1.40E-11	10.9
**SMU_1641c**		conserved hypothetical protein	1.04	2.06	2.45E-06	5.6
**SMU_1070c**		conserved hypothetical protein	1.04	2.06	3.50E-11	10.5
**SMU_932**		conserved hypothetical protein	1.03	2.05	1.02E-07	7.0
**SMU_2096c**		conserved hypothetical protein	1.03	2.05	5.31E-09	8.3
**SMU_1067c**		ABC transporter, permease protein	1.02	2.03	1.69E-11	10.8
**SMU_1348c**			1.02	2.03	2.68E-10	9.6
**SMU_1617**	*era*	GTP-binding protein era homolog.	1.02	2.02	1.08E-09	9.0
**SMU_340**		50S ribosomal protein L34	1.01	2.01	4.33E-13	12.4
**SMU_1904c**		hypothetical protein	-1.00	0.50	1.42E-09	8.8
**SMU_145**		major facilitator superfamily transporter, efflux protein	-1.00	0.50	3.33E-11	10.5
**SMU_300**	*tgt*	tRNA-guanine transglycosylase; queuine tRNA-ribosyltransferase	-1.00	0.50	1.24E-13	12.9
**SMU_1999c**		glutamate--cysteine ligase	-1.00	0.50	2.56E-16	15.6
**SMU_1390**		conserved hypothetical protein	-1.01	0.50	1.59E-10	9.8
**SMU_2038**	*pttB*	phosphotransferase system, trehalose-specific IIBC component (EIIBC-tre)	-1.02	0.49	5.93E-17	16.2
**SMU_301**		conserved hypothetical protein	-1.02	0.49	3.30E-13	12.5
**SMU_1003**	*gid*	glucose-inhibited division protein	-1.02	0.49	7.33E-17	16.1
**SMU_256**	*oppB*	oligopeptide ABC transporter, permease	-1.03	0.49	2.68E-10	9.6
**SMU_1637c**		hypothetical protein	-1.04	0.49	5.93E-11	10.2
**SMU_532**	*trpE*	anthranilate synthase, component I	-1.04	0.49	2.65E-14	13.6
**SMU_887**	*galT*	galactose-1-phosphate uridylyltransferase	-1.06	0.48	1.01E-09	9.0
**SMU_892**	*hsdS*	type I restriction-modification system specificity determinant	-1.06	0.48	5.68E-14	13.2
**SMU_1954**	*groEL*	chaperonin GroEL	-1.07	0.48	6.14E-09	8.2
**SMU_1511c**		acetyltransferase; possible transcriptional repressor	-1.08	0.47	6.62E-16	15.2
**SMU_1955**	*groES*	co-chaperonin 10kDa	-1.09	0.47	2.88E-10	9.5
**SMU_441**		transcriptional regulator	-1.09	0.47	1.23E-08	7.9

**SMU_1463c**		conserved hypothetical protein, NIF3-related	-1.09	0.47	4.69E-12	11.3
**SMU_302**		conserved hypothetical protein	-1.10	0.47	1.65E-12	11.8
**SMU_915c**		conserved hypothetical protein	-1.11	0.46	2.41E-07	6.6
**SMU_872**		fructose-specific PTS system enzyme IIBC component	-1.11	0.46	8.70E-11	10.1
**SMU_378**		hypothetical protein	-1.12	0.46	7.36979E-05	4.1
**SMU_558**		isoleucine-tRNA synthetase	-1.13	0.46	3.03E-15	14.5
**SMU_886**	*galK*	galactokinase	-1.13	0.46	1.02839E-06	6.0
**SMU_856**	*pyrR*	bifunctional protein: pyrimidine operon regulatory protein and uracil phosphoribosyltransferase	-1.14	0.45	4.07E-08	7.4
**SMU_268**	*purA*	adenylosuccinate synthetase	-1.14	0.45	1.08E-13	13.0
**SMU_97**	*pyrG*	CTP synthetase	-1.15	0.45	1.20E-10	9.9
**SMU_1905c**		hypothetical protein	-1.16	0.45	2.18207E-06	5.7
**SMU_893**		anticodon nuclease	-1.18	0.44	4.68E-07	6.3
**SMU_1909c**		hypothetical protein	-1.19	0.44	7.75E-08	7.1
**SMU_438c**		(R)-2-hydroxyglutaryl-CoA dehydratase activator-related protein	-1.20	0.44	3.92E-10	9.4
**SMU_100**		sorbose PTS system, IIB component	-1.21	0.43	2.22E-09	8.7
**SMU_732**		conserved hypothetical protein	-1.21	0.43	6.44E-13	12.2
**SMU_1912c**		hypothetical protein	-1.24	0.42	7.42E-09	8.1
**SMU_440**		hypothetical protein	-1.24	0.42	4.74E-11	10.3
**SMU_957**		50S ribosomal protein L10	-1.26	0.42	4.44E-12	11.4
**SMU_153**		hypothetical protein	-1.27	0.41	1.04E-07	7.0
**SMU_457**		hypothetical protein	-1.28	0.41	1.17E-07	6.9
**SMU_1124**	*pdp*	pyrimidine-nucleoside phosphorylase	-1.28	0.41	2.21E-15	14.7
**SMU_1595**	*cah*	carbonic anhydrase (carbonate dehydratase)	-1.29	0.41	1.70E-13	12.8
**SMU_1907**		hypothetical protein	-1.29	0.41	2.39E-07	6.6
**SMU_1224**	*pyrK*	dihydroorotate dehydrogenase electron transfer subunit	-1.30	0.41	8.30E-09	8.1
**SMU_1910c**		hypothetical protein	-1.32	0.40	1.32E-10	9.9
**SMU_279**		hypothetical protein	-1.33	0.40	2.50E-08	7.6
**SMU_1125c**		conserved hypothetical protein	-1.34	0.40	1.19E-13	12.9
**SMU_152**		hypothetical protein	-1.34	0.39	5.49605E-06	5.3
**SMU_83**	*dnaJ*	co-chaperone protein DnaJ	-1.36	0.39	8.08E-10	9.1
**SMU_1487**		conserved hypothetical protein	-1.37	0.39	3.93E-07	6.4
**SMU_910**	*gtfD*	glucosyltransferase-S	-1.37	0.39	5.03E-16	15.3
**SMU_1223**	*pyrDB*	dihydroorotate dehydrogenase	-1.38	0.38	3.38E-10	9.5
**SMU_1554c**		conserved hypothetical protein	-1.38	0.38	5.00062E-06	5.3
**SMU_1913c**		hypothetical protein; immunity protein, BLpL-like	-1.40	0.38	5.05E-10	9.3
**SMU_2037**	*treA*	trehalose-6-phosphate hydrolase	-1.41	0.38	1.53E-11	10.8

**SMU_151**		non-lantibiotic mutacin IV B	-1.42	0.37	3.98E-07	6.4
**SMU_241c**		amino acid ABC transporter, ATP-binding protein	-1.43	0.37	9.68E-13	12.0
**SMU_1816c**		transposon-related, maturase-related protein fragment	-1.45	0.37	1.23483E-06	5.9
**SMU_859**	*pyrA*	carbamoyl-phosphate synthase,small subunit	-1.50	0.35	2.95E-10	9.5
**SMU_150**		non-lantibiotic mutacin IV A	-1.53	0.35	1.41E-07	6.8
**SMU_242c**		glutamine ABC transporter, solute binding protein	-1.53	0.35	1.33E-08	7.9
**SMU_2146c**		conserved hypothetical protein	-1.54	0.35	1.30E-16	15.9
**SMU_1879**		mannose PTS system component IID	-1.58	0.33	9.56E-09	8.0
**SMU_496**	*cysK*	cysteine synthetase A	-1.59	0.33	5.00E-12	11.3
**SMU_857**		xanthine/uracil permease	-1.60	0.33	4.66E-15	14.3
**SMU_1878**	*ptnC*	mannose PTS system component IIC	-1.60	0.33	1.04E-08	8.0
**SMU_1877**	*ptnA*	mannose PTS system component IIAB	-1.63	0.32	2.17E-11	10.7
**SMU_871**	*pfkB*	fructose-1-phosphate kinase	-1.63	0.32	1.85E-08	7.7
**SMU_858**	*pyrB*	aspartate transcarbamoylase	-1.66	0.32	8.91E-14	13.1
**SMU_1961c**		fructose-specific Enzyme IIA component	-1.67	0.31	1.08E-08	8.0
**SMU_1286c**		multidrug resistance permease	-1.67	0.31	2.79E-12	11.6
**SMU_860**	*pyrAB*	carbamoyl-phosphate synthase, large subunit	-1.76	0.30	1.80E-07	6.7
**SMU_80**	*hrcA*	heat-inducible transcription repressor	-1.85	0.28	3.83E-15	14.4
**SMU_1908c**		hypothetical protein	-1.86	0.28	4.26E-11	10.4
**SMU_1958c**		fructose-specific Enzyme IIC component	-2.14	0.23	7.17E-08	7.1
**SMU_81**	*grpE*	co-chaperone protein GrpE	-2.23	0.21	8.49E-09	8.1
**SMU_870**		lactose phosphotransferase system repressor/transcriptional repressor of the fructose operon	-2.24	0.21	3.27E-08	7.5
**SMU_1956c**		conserved hypothetical protein	-2.28	0.21	1.52E-06	5.8
**SMU_1957**		fructose-specific Enzyme IID component	-2.29	0.20	1.36E-06	5.9
**SMU_148**	*adhE*	alcohol-acetaldehyde dehydrogenase	-2.39	0.19	1.51E-13	12.8

Supplemental Table 3. Distribution of upregulated DEGs (from monoculture growth) between *Streptococcus mutans* UA159 grown in quadculture (*S. mutans*, *Streptococcus gordonii* DL1, *Streptococcus oralis* 34, and *Streptococcus sanguinis* SK36; GSE209925; doi: 10.1177/00220345221145906) vs with *Streptococcus mitis* ATCC 49456 in coculture.

Red – specific to quadculture
Blue – specific to coculture w/ *S. mitis*
Purple – common between both conditions

Gene ID	Gene Name	Gene Description	Upregulated in Quadculture?	Upregulated with *S. mitis*?
SMU_05		conserved hypothetical protein		
SMU_40		conserved hypothetical protein		
SMU_41		hypothetical protein (protein)		
SMU_55		hypothetical protein		
SMU_92c		transposase fragment		
SMU_93c		transposase fragment		
SMU_94c		transposase fragment		
SMU_99	*fbaA*	fructose-bisphosphate aldolase		
SMU_120		50S ribosomal protein L28		
SMU_154		30S ribosomal protein S15		
SMU_169		50S ribosomal protein L13		
SMU_170		30S ribosomal protein S9		
SMU_184	*sloC*	ABC transporter element, iron predicted binding protein		
SMU_185		hypothetical protein		
SMU_186	*sloR*	metal-dependent transcriptional regulator (probable DtxR homolog)		
SMU_191c		phage-related integrase		
SMU_193c		conserved hypothetical protein		
SMU_194c		conserved hypothetical protein, phage-related		
SMU_195c		hypothetical protein		
SMU_196c		immunogenic secreted protein (transfer protein)		
SMU_197c		hypothetical protein		
SMU_198c		conjugative transposon protein		
SMU_199c		hypothetical protein		
SMU_200c		hypothetical protein		
SMU_201c		conserved hypothetical protein		
SMU_202c		conserved hypothetical protein/Streptococcus-specific protein		
SMU_204c		hypothetical protein		

SMU_205c		conserved hypothetical protein
SMU_206c		hypothetical protein
SMU_207c		transcriptional regulator
SMU_208c		conserved hypothetical protein, FtsK/SpoIIIE family
SMU_209c		hypothetical protein
SMU_210c		hypothetical protein
SMU_211c		hypothetical protein
SMU_212c		hypothetical protein
SMU_213c		hypothetical protein
SMU_215c		hypothetical protein
SMU_216c		hypothetical protein
SMU_217c		conserved hypothetical protein; Streptococcus-specific protein
SMU_239c		conserved hypothetical protein
SMU_241c		amino acid ABC transporter, ATP-binding protein
SMU_246	*rgpG*	polysaccharide biosynthesis protein, glycosyl transferase family 4
SMU_299c		bacteriocin peptide precursor
SMU_340		50S ribosomal protein L34
SMU_357		30S ribosomal protein S12
SMU_358		30S ribosomal protein S7
SMU_360	*gapC*	glyceraldehyde-3-phosphate dehydrogenase; plasmin receptor
SMU_361	*pgk*	phosphoglycerate kinase
SMU_367		Streptococcus-specific protein; similar to glucan-binding protein
SMU_402	*pfl*	pyruvate formate-lyase
SMU_411c		Streptococcus-specific protein
SMU_412c		Hit-like protein
SMU_453		S-adenosyl-methyltransferase, MraW family
SMU_527		conserved hypothetical protein
SMU_528c		conserved hypothetical protein
SMU_540	*dpr*	peroxide resistance protein / iron binding protein
SMU_589		histone-like DNA-binding protein
SMU_591c		conserved hypothetical protein
SMU_600c		conserved hypothetical protein
SMU_635		conserved hypothetical protein
SMU_636		glucosamine-6-phosphate isomerase
SMU_637c		hypothetical protein
SMU_671	*citZ*	citrate synthase

SMU_672	*idh*	isocitrate dehydrogenase
SMU_698		50S ribosomal protein L35
SMU_699		50S ribosomal protein L20
SMU_714		translation elongation factor Tu
SMU_739c		hypothetical protein
SMU_753		conserved hypothetical protein
SMU_766		transposase
SMU_769		conserved hypothetical protein
SMU_770c		manganese transporter/possible HitA ferric iron-binding periplasmic protein
SMU_838	*gshR*	glutathione reductase
SMU_849		50S ribosomal protein L27
SMU_865		30S ribosomal protein S16
SMU_911c		hypothetical protein
SMU_924	*tpx*	thiol peroxidase
SMU_925		bacteriocin immunity protein
SMU_932		conserved hypothetical protein
SMU_933		amino acid ABC transporter, amino acid substrate-binding protein
SMU_934		amino acid ABC transporter, permease protein
SMU_935		amino acid ABC transporter, permease protein
SMU_936		amino acid ABC transporter, ATP-binding protein
SMU_940c		hemolysin III-related protein
SMU_941c		conserved hypothetical protein
SMU_943c		hydroxymethylglutaryl-CoA synthase
SMU_961		macrophage infectivity potentiator-related protein
SMU_962		acyl-CoA dehydrogenase
SMU_981	*bglB1*	beta-glucosidase, BglB protein
SMU_995		ferrichrome ABC transporter (permease)
SMU_996		ABC transporter, permease protein;possible ferrichrome transport system
SMU_997		inorganic ion ABC transporter,ATP-binding protein; possible ferrichrome transport system
SMU_998		ABC transporter, ferrichrome-binding protein
SMU_1004	*gtfB*	glucosyltransferase-I
SMU_1036		conserved hypothetical protein
SMU_1062	*opuAb*	glycine-betaine binding ABC transporter permease
SMU_1063	*opuAa*	amino acid ABC transporter, ATP-binding protein
SMU_1067c		ABC transporter, permease protein
SMU_1068c		ABC transporter, ATP-binding protein

SMU_1070c		conserved hypothetical protein
SMU_1072c		acyltransferase
SMU_1091	*wapE*	hypothetical protein (possible cell wall protein)
SMU_1131c		hypothetical protein
SMU_1173	*cysD*	O-acetylhomoserine sulfhydrylase
SMU_1175		sodium:alanine (or glycine) symporter
SMU_1182	*mtlD*	mannitol 1-phosphate 5-dehydrogenase
SMU_1183	*mtlA2*	phosphotransferase system enzyme II
SMU_1184c		transcriptional regulator
SMU_1189c		conserved hypothetical protein
SMU_1197		conserved hypothetical protein
SMU_1247	*eno*	enolase
SMU_1322	*budC*	acetoin reductase
SMU_1335c		enoyl-acyl carrier protein(ACP) reductase; dioxygenase related to 2-nitropropane dioxygenase
SMU_1336	*pksD*	conserved hypothetical protein
SMU_1337c		alpha/beta superfamily hydrolases
SMU_1339	*bacD*	bacitracin synthetase; surfactin synthetase
SMU_1340	*bacA2*	bacitracin synthetase 1/ tyrocidin synthetase III
SMU_1341c		gramicidin S synthase/mycosubtilin synthetase chain mycB
SMU_1342	*bacA1*	bacitracin synthetase
SMU_1343c		polyketide synthase
SMU_1344c		malonyl CoA-acyl carrier protein transacylase
SMU_1345c		peptide synthetase similar to mycA
SMU_1346	*bacT*	thioesterase II-like protein
SMU_1347c		permease
SMU_1348c		ABC transporter
SMU_1365c		permease
SMU_1366c		ABC transporter
SMU_1379		hypothetical protein
SMU_1395c		hypothetical protein
SMU_1396	*gbpC*	glucan-binding protein C
SMU_1467	*apt*	adenine phosphoribosyltransferase
SMU_1470c		conserved hypothetical protein
SMU_1587c		hypothetical protein
SMU_1602		NAD(P)H-flavin oxidoreductase
SMU_1603	*lguL*	lactoylglutathione lyase
SMU_1617	*era*	GTP-binding protein era homolog.
SMU_1626	*rl1*	50S ribosomal protein L1

SMU_1627	*rl11*	50S ribosomal protein L11
SMU_1641c		conserved hypothetical protein
SMU_1649	*exoA*	exodeoxyribonuclease III/ Smx nuclease
SMU_1657c		nitrogen regulatory protein PII
SMU_1658	*nrgA*	ammonium transporter, NrgA protein
SMU_1680c		hypothetical protein
SMU_1682c		conserved hypothetical protein (possible intracellular protease)
SMU_1743	*acp*	acyl carrier protein
SMU_1750c		hypothetical protein
SMU_1752c		hypothetical protein
SMU_1753c		conserved hypothetical protein
SMU_1754c		conserved hypothetical protein
SMU_1755c		conserved hypothetical protein
SMU_1757c		conserved hypothetical protein
SMU_1758c		conserved hypothetical protein
SMU_1760c		conserved hypothetical protein
SMU_1761c		conserved hypothetical protein
SMU_1762c		conserved hypothetical protein
SMU_1763c		conserved hypothetical protein
SMU_1764c		conserved hypothetical protein
SMU_1781		conserved hypothetical protein
SMU_1782		conserved hypothetical protein
SMU_1802c		conserved hypothetical protein
SMU_1858	*rs18*	30S ribosomal protein S18
SMU_1860	*rs6*	30S ribosomal protein S6
SMU_1862		hypothetical protein
SMU_1888		transposase fragment
SMU_1889c		hypothetical protein (possible relation to bacteriocin BlpU)
SMU_1895c		hypothetical protein
SMU_1896c		hypothetical protein
SMU_1897		ABC transporter, ATP-binding protein; similar to BlpA
SMU_1898		ABC transporter, ATP-binding and permease element
SMU_1915	*comC*	S. mutans specific competence stimulating peptide, precursor
SMU_1945		conserved hypothetical protein
SMU_1946		conserved hypothetical protein
SMU_1988c		probable DNA binding protein
SMU_2002	*rs11*	30S ribosomal protein S11

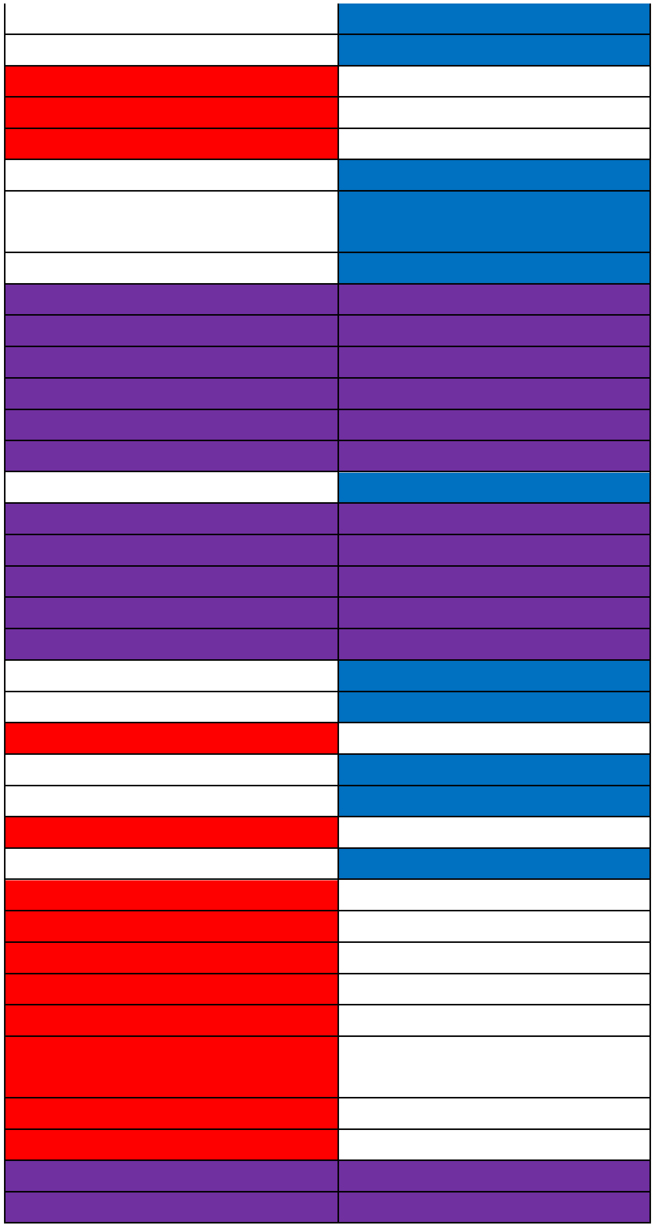

SMU_2009	*rs5*	30S ribosomal protein S5		
SMU_2010	*rl18*	50S ribosomal protein L18		
SMU_2016	*rl24*	50S ribosomal protein L24		
SMU_2017	*rl14*	50S ribosomal protein L14		
SMU_2018	*rs17*	30S ribosomal protein S17		
SMU_2019	*rl29*	50s ribosomal protein L29		
SMU_2020	*rl16*	50S ribosomal protein L16		
SMU_2021	*rs3*	30S ribosomal protein S3		
SMU_2022	*rl22*	50S ribosomal protein L22		
SMU_2023c		30S ribosomal protein S19, C-terminal fragment		
SMU_2025	*rl3*	50S ribosomal protein L3		
SMU_2026c		30S ribosomal protein S10 fragment		
SMU_2027		transcriptional regulator/repressor		
SMU_2028	*sacB*	fructosyltransferase		
SMU_2057c		cadmium-efflux ATPase, E1-E2 (heavy metal-transporting ATPase)		
SMU_2093	*argR*	arginine repressor		
SMU_2096c		conserved hypothetical protein		
SMU_2104a		50S ribosomal protein L32		
SMU_2116	*opuCa*	glycine betaine / carnitine / choline ABC transporter, ATP-binding protein, opuCA		
SMU_2117	*opuCb*	glycine betaine / carnitine / choline ABC transporter permease		
SMU_2118	*opuCc*	glycine betaine/carnitine/choline ABC transporter, substrate-binding protein		
SMU_2119	*opuCd*	ABC transport betaine/carnitine/choline permease		
SMU_2146c		conserved hypothetical protein		
SMU_2147c		conserved hypothetical protein		

Supplemental Table 4. Distribution of downregulated DEGs (from monoculture growth) between *Streptococcus mutans* UA159 grown in quadculture (*S. mutans*, *Streptococcus gordonii* DL1, *Streptococcus oralis* 34, and *Streptococcus sanguinis* SK36; GSE209925; doi: 10.1177/00220345221145906) vs with *Streptococcus mitis* ATCC 49456 in coculture.

Red – specific to quadculture
Blue – specific to coculture w/ *S. mitis*
Purple – common between both conditions

Gene ID	Gene Name	Gene Description	Downregulated in Quadculture?	Downregulated with *S. mitis*?
SMU_21	*mreD*	cell shape-determining protein MreD		
SMU_29		phosphoribosylaminoimidazole-succinocarboxamide synthase		
SMU_78	*fruA*	fructan hydrolase; exo-beta-D-fructosidase		
SMU_80	*hrcA*	heat-inducible transcription repressor		
SMU_81	*grpE*	co-chaperone protein GrpE		
SMU_83	*dnaJ*	co-chaperone protein DnaJ		
SMU_97	*pyrG*	CTP synthetase		
SMU_100		sorbose PTS system, IIB component		
SMU_145		major facilitator superfamily transporter, efflux protein		
SMU_148	*adhE*	alcohol-acetaldehyde dehydrogenase		
SMU_150		non-lantibiotic mutacin IV A		
SMU_151		non-lantibiotic mutacin IV B		
SMU_152		hypothetical protein		
SMU_153		hypothetical protein		
SMU_231	*ilvB*	acetolactate synthase, large subunit (AHAS)		
SMU_233	*ilvC*	ketol-acid reductoisomerase		
SMU_241c		amino acid ABC transporter, ATP-binding protein		
SMU_242c		glutamine ABC transporter, solute binding protein		
SMU_256	*oppB*	oligopeptide ABC transporter, permease		
SMU_268	*purA*	adenylosuccinate synthetase		
SMU_279		hypothetical protein		
SMU_300	*tgt*	tRNA-guanine transglycosylase; queuine tRNA-ribosyltransferase		
SMU_301		conserved hypothetical protein		
SMU_302		conserved hypothetical protein		
SMU_378		hypothetical protein		
SMU_419		conserved hypothetical protein		
SMU_423		possible bacteriocin		

SMU_438c		(R)-2-hydroxyglutaryl-CoA dehydratase activator-related protein
SMU_440		hypothetical protein
SMU_441		transcriptional regulator
SMU_454	*ftsL*	cell division protein
SMU_457		hypothetical protein
SMU_496	*cysK*	cysteine synthetase A
SMU_525		ABC transporter ATP-binding / permease protein
SMU_532	*trpE*	anthranilate synthase, component I
SMU_558		isoleucine-tRNA synthetase
SMU_574c		effector of murein hydrolase
SMU_575c		murein hydrolase regulator
SMU_609		cell wall protein precursor
SMU_691	*pepT*	peptidase T (tripeptidase)
SMU_732		conserved hypothetical protein
SMU_856	*pyrR*	bifunctional protein: pyrimidine operon regulatory protein and uracil phosphoribosyltransferase
SMU_857		xanthine/uracil permease
SMU_858	*pyrB*	aspartate transcarbamoylase
SMU_859	*pyrA*	carbamoyl-phosphate synthase,small subunit
SMU_860	*pyrAB*	carbamoyl-phosphate synthase, large subunit
SMU_867	*rimM*	16S rRNA processing protein
SMU_870		lactose phosphotransferase system repressor/transcriptional repressor of the fructose operon
SMU_871	*pfkB*	fructose-1-phosphate kinase
SMU_872		fructose-specific PTS system enzyme IIBC component
SMU_886	*galK*	galactokinase
SMU_887	*galT*	galactose-1-phosphate uridylyltransferase
SMU_892	*hsdS*	type I restriction-modification system specificity determinant
SMU_893		anticodon nuclease
SMU_910	*gtfD*	glucosyltransferase-S
SMU_915c		conserved hypothetical protein
SMU_930c		transcriptional regulator
SMU_940c		hemolysin III-related protein
SMU_941c		conserved hypothetical protein
SMU_957		50S ribosomal protein L10
SMU_1003	*gid*	glucose-inhibited division protein
SMU_1124	*pdp*	pyrimidine-nucleoside phosphorylase

SMU_1125c		conserved hypothetical protein
SMU_1134c		phosphate ABC transporter, ATP-binding protein
SMU_1177c		amino acid ABC transporter, amino acid-binding protein
SMU_1178c		amino acid ABC transporter, ATP-binding protein
SMU_1223	*pyrDB*	dihydroorotate dehydrogenase
SMU_1224	*pyrK*	dihydroorotate dehydrogenase electron transfer subunit
SMU_1237c		hypothetical protein
SMU_1246c		transcriptional regulator (TetR/AcrR family)
SMU_1254		phosphatase
SMU_1271	*hisG*	ATP phosphoribosyltransferase
SMU_1286c		multidrug resistance permease
SMU_1334	*sfp*	phosphopantetheinyl transferase
SMU_1335c		enoyl-acyl carrier protein(ACP) reductase; dioxygenase related to 2-nitropropane dioxygenase
SMU_1337c		alpha/beta superfamily hydrolases
SMU_1338c		ABC transport macrolide permease
SMU_1339	*bacD*	bacitracin synthetase; surfactin synthetase
SMU_1340	*bacA2*	bacitracin synthetase 1/ tyrocidin synthetase III
SMU_1341c		gramicidin S synthase/mycosubtilin synthetase chain mycB
SMU_1342	*bacA1*	bacitracin synthetase
SMU_1343c		polyketide synthase
SMU_1344c		malonyl CoA-acyl carrier protein transacylase
SMU_1345c		peptide synthetase similar to mycA
SMU_1346	*bacT*	thioesterase II-like protein
SMU_1348c		ABC transporter
SMU_1367c		conserved hypothetical protein
SMU_1390		conserved hypothetical protein
SMU_1402c		conserved hypothetical protein
SMU_1404c		conserved hypothetical protein
SMU_1435c		hypothetical protein
SMU_1463c		conserved hypothetical protein, NIF3-related
SMU_1464c		conserved hypothetical protein
SMU_1487		conserved hypothetical protein
SMU_1511c		acetyltransferase; possible transcriptional repressor
SMU_1532	*atpF*	ATPase, b subunit
SMU_1554c		conserved hypothetical protein
SMU_1595	*cah*	carbonic anhydrase (carbonate dehydratase)
SMU_1615c		conserved hypothetical protein
SMU_1637c		hypothetical protein

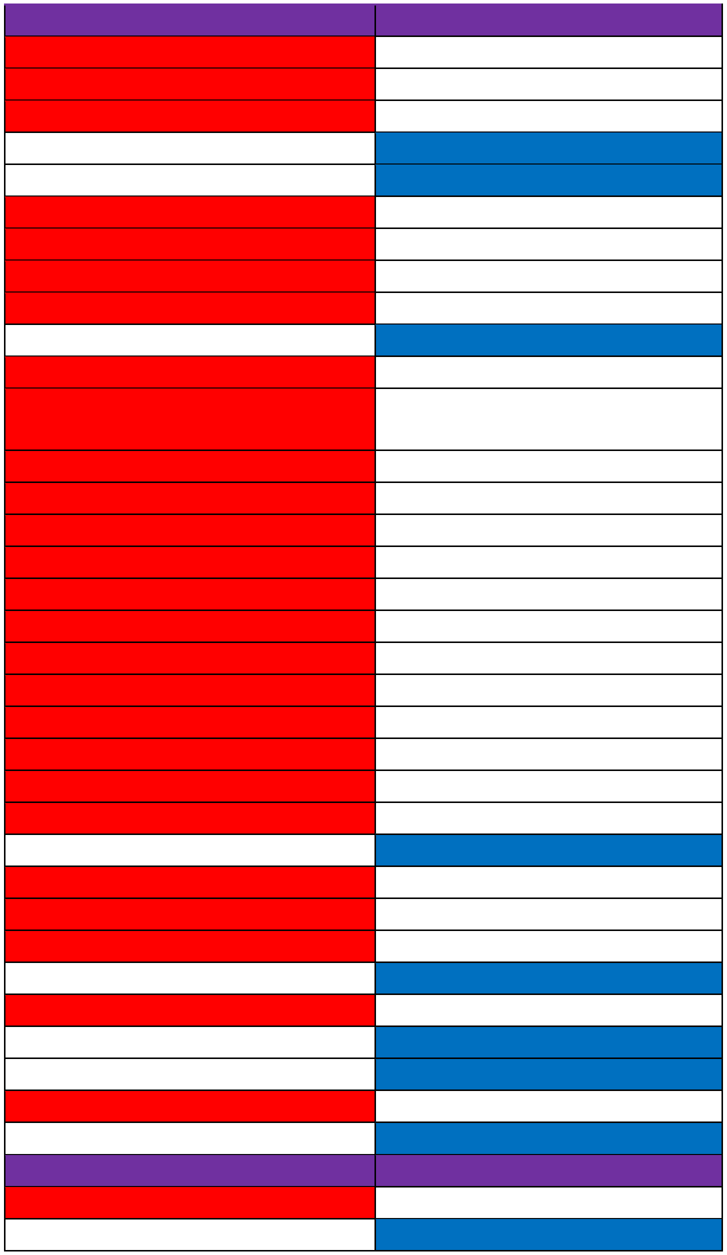

SMU_1732c		conserved hypothetical protein		
SMU_1734	*accA*	acetyl-CoA carboxylase alpha subunit		
SMU_1735	*accD*	acetyl-CoA carboxylase beta subunit		
SMU_1816c		transposon-related, maturase-related protein fragment		
SMU_1877	*ptnA*	mannose PTS system component IIAB		
SMU_1878	*ptnC*	mannose PTS system component IIC		
SMU_1879		mannose PTS system component IID		
SMU_1903c		hypothetical protein		
SMU_1904c		hypothetical protein		
SMU_1905c		hypothetical protein		
SMU_1906c		bacteriocin-related protein		
SMU_1907		hypothetical protein		
SMU_1908c		hypothetical protein		
SMU_1909c		hypothetical protein		
SMU_1910c		hypothetical protein		
SMU_1912c		hypothetical protein		
SMU_1913c		hypothetical protein; immunity protein, BLpL-like		
SMU_1914c		bacteriocin protein, BlpO-like		
SMU_1941	*atmB*	ABC transporter solute-binding protein		
SMU_1954	*groEL*	chaperonin GroEL		
SMU_1955	*groES*	co-chaperonin 10kDa		
SMU_1956c		conserved hypothetical protein		
SMU_1957		fructose-specific Enzyme IID component		
SMU_1958c		fructose-specific Enzyme IIC component		
SMU_1960c		fructose-specific Enzyme IIB component		
SMU_1961c		fructose-specific Enzyme IIA component		
SMU_1999c		glutamate--cysteine ligase		
SMU_2019	*rl29*	50s ribosomal protein L29		
SMU_2020	*rl16*	50S ribosomal protein L16		
SMU_2037	*treA*	trehalose-6-phosphate hydrolase		
SMU_2038	*pttB*	phosphotransferase system, trehalose-specific IIBC component (EIIBC-tre)		
SMU_2053c		hypothetical protein		
SMU_2146c		conserved hypothetical protein		

Supplemental Table 5. Bacterial strains used in this study.

Species	Strain	Genotype or description	Antibiotic resistance	Reference or source
Actinomyces oris	WVU627			Lab Stock
Streptococcus sp.	A12			Lab Stock
Streptococcus cristatus	ATCC 51100			Lab Stock
Streptococcus gordonii	DL1			Lab Stock
Streptococcus mits	ATCC 49456			Lab Stock
	49456 Δ*spxB*	Allelic exchange of pyruvate oxidase gene (SM12261_RS06360, SM12261_1237) with an antibiotic resistant cassette	Erythromycin	This manuscript
	B6			Lab Stock
	SK306			Lab Stock
	SK569			Lab Stock
Streptococcus mutans	UA159 (SMU159)			Lab stock
	1ID3 (SMU009)			Lab stock
	15JP3 (SMU020)			Lab stock
	2VS1 (SMU041)			Lab stock
	NVAB (SMU053)			Lab stock
	NMT4863 (SMU057)			Lab stock
	A19 (SMU058)			Lab stock
	N66 (SMU076)			Lab stock
	SF1 (SMU080)			Lab stock
	SM6 (SMU082)			Lab stock
	U2A (SMU086)			Lab stock
	NLML1 (SMU089)			Lab stock
	21 (SMU093)			Lab stock
	SM1 (SMU098)			Lab stock
	U2B (SMU101)			Lab stock
	SA41 (SMU104)			Lab stock
	SF12 (SMU105)			Lab stock
	R221 (SMU107)			Lab stock
	M230 (SMU108)			Lab stock
	OMZ175 (SMU109)			Lab stock
	pMZ- / UA159	UA159 with pMZ plasmid integrated into genome. Serves as a "marked" *S. mutans* strain during colony forming unit (CFU) assays	Kanamycin	Sheilds et al. 2019, Appl Environ Microbiol.
	pMZ-P*veg*::*gfp* / UA159	UA159 with pMZ plasmid integrated into genome harboring constitutively-active *gfp*. Serves as GFP(+) strain used in coculture microscopy experiments	Kanamycin	Sheilds et al. 2019, Appl Environ Microbiol.
Streptococcus oralis	34			Lab Stock
Streptococcus sanguinis	SK36			Lab Stock
Streptococcus sobrinus	6715			Lab Stock

Supplemental Table 6. Concentration* of inoculated bacterial strains during competitive competitions.

* Concentrations were determined based on the growth rate of individual species, such that the initial growth rate and entrance into exponential growth phase is matched to that of *Streptococcus mutans* pMZ-P*veg*::*gfp* / UA159 strain.

from doi: 10.1128/msphere.00771-23

Strain Name	Concentration of Competitor	***S. mutans* / *S. mitis* Concentration**
S. sp. A12	0.5x	1x
S. cristatus ATCC 51100	10x	1x
S. gordonii DL1	10% solution (1:10 dilution); 1x	1x
S. mitis ATCC 49456	1x	1x
S. oralis 34	1x	1x
S. sanguinis SK36	1x	1x
S. sobrinus 6715	2x	1x

Supplemental Table 7. Primers used in this study.

***Bold and underline** denotes BamHI cut site used in the PCR ligation mutagenesis approach

Primer Name	Primer Sequence (5' - 3') *	Tm
mitis-spxB-A	TGC CAT TGC CGA TCA GAT CGG	60.1
mitis-spxB-B	CAT **GGA TCC** TTG CTG CAG ATG CAG	60.4
mitis-spxB-C	CGA **GGA TCC** AGA ACT CGT ACC ATT CCG	61.8
mitis-spxB-D	GTA ATC GGG ATC ATT GCT GGT AGT GC	59.5

Supplemental Table 8. RNA-Seq read counts assigned to each species. The first table expresses total number of raw read counts for each species per library, wile the second table is expressed in % of counts within the library.

Sample	Sample Name	*S. mutans* Counts	*S. mitis* Counts	Unassigned Counts	Total Counts
1	SMU_Mono_TYG_1	28,039,445	-	197,509	**28,236,954**
2	SMU_Mono_TYG_2	26,483,264	-	203,762	**26,687,026**
3	SMU_Mono_TYG_3	24,111,846	-	200,892	**24,312,738**
4	Mitis_Mono_TYG_1	-	26,330,498	234,772	**26,565,270**
5	Mitis_Mono_TYG_2	-	22,977,370	148,578	**23,125,948**
6	Mitis_Mono_TYG_3	-	25,646,326	146,406	**25,792,732**
7	SMU_CoMitis_TYG_1	16,051,834	30,664,536	98,648	**45,632,834**
8	SMU_CoMitis_TYG_2	15,442,671	31,052,053	97,537	**45,571,812**
9	SMU_CoMitis_TYG_3	15,502,895	29,075,512	94,681	**43,596,136**
**Sample**	**Sample Name**	**SMU % Counts**	**So34 % Counts**	**Unassigned % Counts**	**Total % Counts**
1	SMU_Mono_TYG_1	99.3%	0.0%	0.7%	100.0%
2	SMU_Mono_TYG_2	99.2%	0.0%	0.8%	100.0%
3	SMU_Mono_TYG_3	99.2%	0.0%	0.8%	100.0%
4	Mitis_Mono_TYG_1	0.0%	99.1%	0.9%	100.0%
5	Mitis_Mono_TYG_2	0.0%	99.4%	0.6%	100.0%
6	Mitis_Mono_TYG_3	0.0%	99.4%	0.6%	100.0%
7	SMU_CoMitis_TYG_1	35.2%	67.2%	0.2%	102.6%
8	SMU_CoMitis_TYG_2	33.9%	68.1%	0.2%	102.2%
9	SMU_CoMitis_TYG_3	35.6%	66.7%	0.2%	102.5%

Supplemental Table 9. Genome files used in this study.

Species	Isolate	Accession	Reference?	Source
Streptococcus mutans	UA159	NC_004350.2	Yes	NCBI GenBank
Streptococcus mitis	NCTC 12261 (ATCC 49456)	CP028414.1	No	NCBI GenBank
